# Spermine: Its Emerging Role in Regulating Drought Stress Responses in Plants

**DOI:** 10.3390/cells10020261

**Published:** 2021-01-28

**Authors:** Md. Mahadi Hasan, Milan Skalicky, Mohammad Shah Jahan, Md. Nazmul Hossain, Zunaira Anwar, Zheng-Fei Nie, Nadiyah M. Alabdallah, Marian Brestic, Vaclav Hejnak, Xiang-Wen Fang

**Affiliations:** 1 State Key Laboratory of Grassland Agro-Ecosystems, School of Life Sciences, Lanzhou University, Lanzhou 730000, China; hasanmahadikau@gmail.com (M.M.H.); niezhf19@lzu.edu.cn (Z.-F.N.); 2 Department of Botany and Plant Physiology, Faculty of Agrobiology, Food and Natural Resources, Czech University of Life Sciences Prague, 16500 Prague, Czech Republic; skalicky@af.czu.cz (M.S.); marian.brestic@uniag.sk (M.B.); hejnak@af.czu.cz (V.H.); 3 Key Laboratory of Southern Vegetable Crop Genetic Improvement in Ministry of Agriculture, College of Horticulture, Nanjing Agricultural University, Nanjing 210095, China; shahjahansau@gmail.com; 4 Department of Horticulture, Sher-e-Bangla Agricultural University, Dhaka 1207, Bangladesh; 5 Key Laboratory of Crop Physiology and Ecology in Southern China, Ministry of Agriculture, Nanjing Agricultural University, Nanjing 210095, China; nnazmul99@gmail.com; 6 Department of Botany, Faculty of Sciences, University of Agriculture Faisalabad, Faisalabad 38000, Pakistan; zunaira96r@yahoo.com; 7 Department of Biology, College of Science, Imam Abdulrahman Bin Faisal University, 383, Dammam 34212, Saudi Arabia; Nmalabdallah@iau.edu.sa; 8 Department of Plant Physiology, Faculty of Agrobiology and Food Resources, Slovak University of Agriculture, 94976 Nitra, Slovakia

**Keywords:** drought, antioxidant enzymes, polyamines, stomata, abscisic acid

## Abstract

In recent years, research on spermine (Spm) has turned up a lot of new information about this essential polyamine, especially as it is able to counteract damage from abiotic stresses. Spm has been shown to protect plants from a variety of environmental insults, but whether it can prevent the adverse effects of drought has not yet been reported. Drought stress increases endogenous Spm in plants and exogenous application of Spm improves the plants’ ability to tolerate drought stress. Spm’s role in enhancing antioxidant defense mechanisms, glyoxalase systems, methylglyoxal (MG) detoxification, and creating tolerance for drought-induced oxidative stress is well documented in plants. However, the influences of enzyme activity and osmoregulation on Spm biosynthesis and metabolism are variable. Spm interacts with other molecules like nitric oxide (NO) and phytohormones such as abscisic acid, salicylic acid, brassinosteroids, and ethylene, to coordinate the reactions necessary for developing drought tolerance. This review focuses on the role of Spm in plants under severe drought stress. We have proposed models to explain how Spm interacts with existing defense mechanisms in plants to improve drought tolerance.

## 1. Introduction

Polyamines (PAs) are water-soluble polycations that have important roles in the normal physiological and developmental functions of plants, as well as in the development of tolerance under conditions of abiotic stress [1,2]. Spermine (Spm), putrescine (Put), and spermidine (Spd) are low-molecular weight polyamines with aliphatic nitrogenous bases that are found in almost all types of living organisms [2]. They serve indispensable functions in physiological and developmental processes such as cell division, embryogenesis, floral emergence, leaf senescence, and responses to abiotic stress [3]. Spm is specifically involved in shoot and root development, floral induction, fruit set, leaf senescence, DNA synthesis, osmolyte balance, chlorophyll protection, gene transcription, and protein translation [4,5,6,7,8,9]. Spm is also crucial for mounting an effective response to environmental stresses such as those caused by drought [10,11,12,13], heavy metals [14,15,16], excessive heat [17], low temperatures [18], and high temperatures [19].

Drought is a major global threat to farming as the resulting stress severely alters key physiological and developmental processes [20,21,22,23,24,25], reducing production by as much as 25% [26]. Long-term drought leads to physiological and metabolic changes in plants including loss of cell turgor, water and mineral imbalances, and photosynthetic abnormalities [27]. However, Spm can significantly enhance plants’ resistance to several environmental stressors, including drought, salt, and heavy metals. Past studies reported that increasing the concentration of endogenous polyamines such as Spm in plants under water deficit conditions significantly increased tolerance [28]. The exogenous application of Spm upregulated the antioxidant systems involving superoxide dismutase (SOD), catalase (CAT), ascorbate peroxidase (APX), monodehydroascorbate reductase (MDHAR), dehydroascorbate reductase (DHAR), glutathione reductase (GR), glutathione S-transferase (GST), and glutathione peroxidase (GPX) [29,30].

We have reviewed the current literature on Spm’s biosynthesis, metabolism, and molecular interactions in response to drought stress in plants along with enhancement of drought stress resistance through regulation of Spm metabolism and external application of Spm. The purpose of this review was to clarify the mechanisms involved in stress resistance and Spm-mediated enhancement of plant tolerance through antioxidant activity and synergy with other molecules in plants under drought stress.

## 2. Spermine Biosynthesis and Metabolism in Plants

Spm biosynthesis is accomplished via two main pathways [20]. In the first pathway, the enzyme arginase converts arginine (arg) into ornithine, which is then transformed into putrescine by the enzyme, ornithine decarboxylase. Putrescine is a precursor of spermine. The second route comprises three pathways, which involve the conversion of arginine into agmatine by two enzymes, agmatine imidohydrolase and carbamoylputrescine amidohydrolase. Subsequently, spermidine synthase converts putrescine into spermidine, which is then transformed into spermine by spermine synthase [31,32,33].

**Figure 1:**
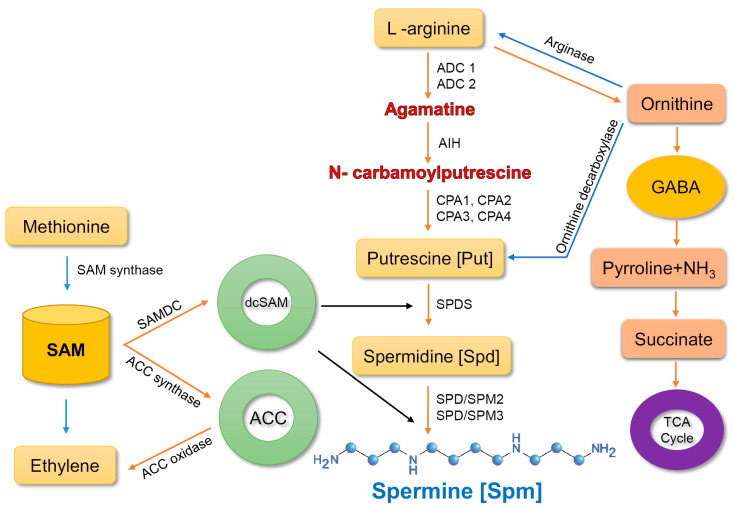
Spermine biosynthesis in plants. ADC, arginine decarboxylase; AIH, agmatine iminohydrolase; CPA-N, carbamoylputrescine amidohydrolase; SPDS, spermidine synthase; SPMS, spermine synthase; GABA, γ-aminobutyric acid; SAM-S, adenosylmethionine; SAMDC-S, adenosylmethionine decarboxylase; dcSAM, decarboxylated S-adenosylmethionine; ACC, 1-aminocyclopropane-1-carboxylic-acid synthase. Arrows represent synthesis and conversion.

In the final reaction, aminopropyl groups are added from decarboxylated S-adenosylmethionine (SAM), which is produced by SAM decarboxylase (SAMDC). These enzymes drive two types of reaction; terminal oxidation and back-conversion reaction. In terminal oxidation of Spm, 4-*N*-(3-aminopropyl)-4-aminobutanal, 1,3-diaminopropane, and H_2_O_2_ are produced. In the back-conversion reaction, Spm is transformed into Spd and Spd into Put, consequently leading to the generation of 3-aminopropanal and H_2_O_2_ [33] (Figure 1).

## 3. Spermine Induced Drought Tolerance in Plants

Low water availability is one of the major abiotic stresses that severely affects plant growth and yield and leads to a decline in defense mechanisms [34]. Adequate soil water for short to long distance transport, osmoregulation, and single cell expansion through cellular membranes is vital for good crop production [35,36]. Drought negatively affects the movement of water in plants, but this can be partly overcome through the opening of membrane channels known as aquaporins (AQPs) that facilitate water permeability [36,37]. To maintain water balance, plants often synthesize polyamines like spermine that stabilize cell membranes and improve water use efficiency [38,39,40]. Recently, Li et al. (2020) [38] reported that Spm helped to maintain water balance under drought stress by increasing expression of the Ca^2+^-dependent AQPs, TrTIP2-1, TrTIP2-2, and TrPIP2-7.

However, the mechanism of spermine-mediated drought tolerance remained unclear. Spm regulates potassium channels and guard cells to control water loss by optimizing stomatal opening and closing [41,42]. Spm can regulate several abscisic acid-related genes, which in turn control stomatal closure, stress-response gene expression, and osmolyte production [43]. A significant positive correlation was seen between spermine levels and grain weight and filling rates in drought-tolerant wheat [44].

**Figure 2:**
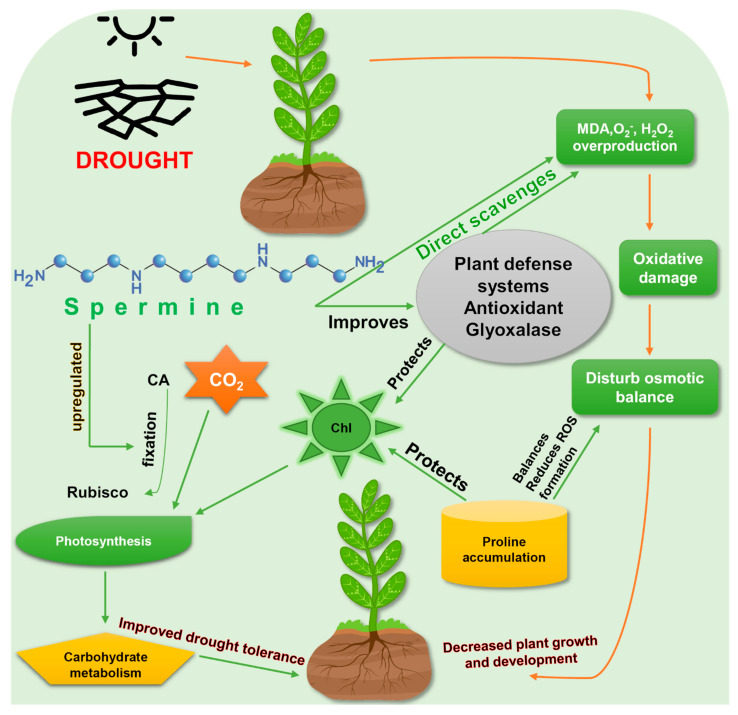
Enhancement of drought-stress tolerance by spermine. Exogenous application of spermine improves the drought tolerance in plants. Rubisco, ribulose-1,5-bisphosphate carboxylase/oxygenase; MDA, malondialdehyde; ROS, reactive oxygen species; Chl, chlorophyll; CO_2,_ carbon dioxide; CA, carbonic anhydrase. Green arrows represent spermine’s actions to reduce drought stress, while red arrows show the direct effects of drought on plants.

Increased production of Spm is a common stress response to drought in several plants such as rice [39], tomato [45], and yellow lupin [46]. Adamipour (2020) [28] found endogenous Spm accumulation in drought affected *Rosa damascene* seedlings and induction of defense mechanisms to mitigate drought stress. It has been confirmed that both endogenously produced and exogenously applied Spm are effective against drought stress [28,39], by enhancing drought-tolerance mechanisms (Figure 2).

Exogenous foliar application of Spm increased survival rate, shoot length and weight, root length and weight, produced greener leaf tissues, and slowed water loss in Bermuda grass (*Cynodon dactylon*) [47]. Photosynthetic efficiency (*F_V_*/*F_M_*) and photosystem II (PSII) activity were found to be higher in Spm-treated plants under drought stress [48]. Levels of osmolytes such as proline and soluble sugars were also increased by spermine. Spm enhanced drought tolerance in creeping bentgrass (*Agrostis stolonifera*) through osmotic adjustment and hormonal regulation. Concentrations of gibberellic acid (GA1, GA4) and Abscisic acid (ABA) in Spm-treated creeping bentgrass were significantly increased under drought stress, which indicates a hormonal connection in Spm’s ability to promote drought tolerance [48] (Table 1).

An increase in Spm has also been associated with drought tolerance in cherry tomatoes [59]. Overexpression of the *DsADC* gene in transgenic rice produced greater drought tolerance through conversion of Put to Spd and Spm [60]. The *Arabidopsis acl5*/*spms* mutant showed hypersensitivity to drought [49]. Liu et al. 2018 [11] treated lettuce plants with 0.1 mM Spm under drought stress induced by 10% PEG and observed significant improvement in morphological and physiological traits. Similar results were seen in mung bean seedlings with higher proline accumulation, osmotic protection, and increased chlorophyll synthesis under drought stress with Spm [29]. In soybean plants under drought stress, 0.2 mM Spm turned out to be the optimal concentration for increasing relative water content (RWC), osmoprotectant concentration, and mineral nutrients [10]. They also found that Spm alleviated drought stress in soybean plants by increasing endogenous spermine biosynthesis [30]. Other scientists reported that exogenous application of Spm to plants positively regulated photosynthetic activity [9,48,61].

Germination of seeds and survival of seedlings under environmental stress is a challenging goal for better crop yield [62]. Several studies have shown that Spm application to seeds is equally effective in promoting germination and early growth of seedlings. The crop yield can be significantly improved by treating seeds with Spm [30]. Seeds treated with Spm produced plants with improved PSII center activity, higher chlorophyll content, and balanced osmolyte accumulation [10]. Together, this body of evidence supports the idea that Spm treatment of seeds or plants can improve drought tolerance and osmoregulation, enhance antioxidant defense, and increase photosynthesis.

## 4. Spermine Activates Antioxidant Response in Plants under Drought Stress

The generation of reactive oxygen species (ROS) occurs in various plant cell compartments such as plasma membranes, peroxisomes, chloroplasts, and mitochondria under normal and stress conditions. Chloroplasts and peroxisomes are the key sites of ROS productions under normal light conditions [63]. Overproduction of ROS in plants is associated with oxidative damage [64,65] and is affected by genotype, the stage of development, and the presence of stresses like drought [66]. Plants adapt to the adverse effects of drought by increasing their antioxidant defenses [67], which include non-enzymatic compounds such as carotenoids, proline, anthocyanin, glycine betaine, α-tocopherols, flavonols, and amino acids and enzymatic antioxidants such as SOD, CAT, APX, MDHAR, DHAR, GR, GST, and GPX [63].

**Figure 3:**
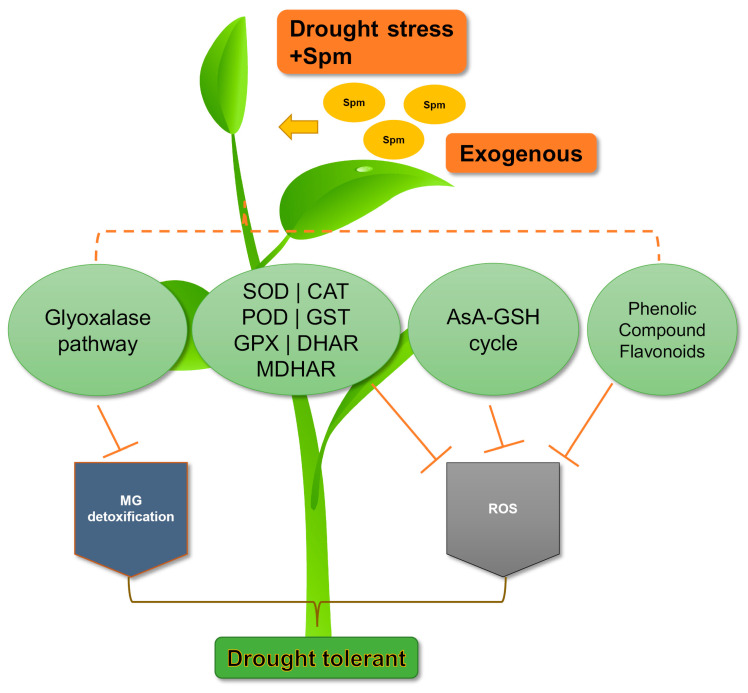
Spermine (Spm)-induced antioxidant defense and glyoxalase system reduces drought stress in plants. The glyoxalase pathway suppresses methylglyoxal (MG) toxicity. Likewise, the antioxidant enzymes, e.g., superoxide dismutase, SOD; catalase, CAT; peroxidase, POD; glutathione S-transferase, GST; glutathione peroxidase, GPX; dehydroascorbate reductase, DHAR, and monodehydroascorbate reductase, MDHAR, and the non-enzymatic compounds, e.g., phenols and flavonoids, ascorbate, AsA, and glutathione suppress the accumulation of ROS.

Spm reduces stress from drought, high temperatures, and heavy metals by upregulating antioxidant enzymes [29]. The elevated concentrations of natural Spm are induced as a part of the antioxidant system under stress conditions [68]. The fruits of the drought-tolerant tomato variety Zarina have high concentrations of endogenous Spm, which upregulates superoxide dismutase (SOD) and catalase (CAT) antioxidant enzyme activity and increases resistance to oxidative stress caused by dehydration [45]. In trifoliate orange seedlings, treatment with 10 µM spermine increased SOD, peroxidase (POD), and APX activity in drought stress compared to non-treated plants [54]. Shi et al. 2013 [47] reported higher SOD, CAT, and POD activity in *Cynodon dactylon* seedlings pretreated with 5 mM Spm under drought stress. Similarly, the treatment of drought-stressed soybean seedlings with Spm increased chlorophyll, carotenoid, and protein levels and improved CAT and SOD activity [57]. Spm application (25 mg/L) with 24-epibrassinolide (0.1 mg/L) enhanced SOD, CAT, APX, MDHAR, DHAR, and GR activity in maize seedlings under drought stress [42]. Shi et al. 2010 [55] concluded that treatment of *Citrus reticulata* seedlings with 1 mM Spm increased the SOD and POD activity under dehydration. In orange plants subjected to combined drought and heat stresses, Spm treatment enhanced CAT, SOD, and POD activity and accelerated the function of heat shock proteins [16]. Together, these findings suggest that Spm promotes resistance to oxidative stress induced by abiotic stressors like drought by activation of glyoxalase and antioxidant pathways (Figure 3).

## 5. Interaction of Spermine with Other Molecules in Drought Tolerance

The polyamine metabolic pathway is closely interconnected with hormones and signaling molecules involved in generating the various stress responses. ABA and NO work together with spermine at the physiological and transcript level to create an appropriate response to drought, particularly with regard to stomatal closure [69]. ABA is an important anti-transpiration molecule that decreases water loss by triggering guard cells to close the stomatal apertures on the leaf surface. It has been reported that Spm and other polyamines also control stomatal opening and closing [42,70].

Transcript profiling has shown that drought triggers expression of the *ADC2*, *SPDS1*, and *SPMS* genes [71] and that the application of ABA induced expression of these genes [72,73]. These findings were verified through tests on ABA-deficient (aba2-3) and ABA-insensitive (abi1-1) mutants under water deficit conditions [71]. The results support the conclusion that the gene expression related to polyamine biosynthesis is regulated by ABA induced in plants exposed to drought [74]. ABA increased endogenous polyamine (Spm) content and both ABA and Spm trigger stomatal closure under drought conditions, thus protecting plants from dehydration [75]. Therefore, we may conclude that polyamines play a significant role in the regulation of stomatal responses by directly or indirectly interacting with ABA [69,74]. Crosstalk between Spm and ABA was observed in *Arabidopsis* overexpressing *SAMDC1*. These transgenic lines displayed high ABA levels because of induction of gene expression for 9-cis-epoxycarotenoid dioxygenase (NECD) [76]. Polyamines also stimulate NO production, which may function in the polyamine-mediated stress response against multiple stressors [77]. Polyamines such as Spm trigger NO production in diverse plant species, suggesting that NO closely interacts with polyamines to mitigate stress [78]. Conversely, polyamines can function as regulators of stomatal closure via the induction of H_2_O_2_ and NO signaling molecules through various pathways [79].

Polyamines (PAs) and ethylene may compete antagonistically for SAM as a common precursor since it is used for ethylene biosynthesis in higher plants. SAM is converted to ACC through the action of 1-aminocyclopropane-1-carboxylate (ACC) synthase, and ACC is oxidized to ethylene [80,81]. PAs play a key role in plant growth and inhibit senescence. In contrast, ethylene enhances senescence and fruit ripening [82]. The ability of PAs to block senescence is well documented [83]. The anti-senescence effect of PAs might be occurring through PA-mediated blocking of ethylene biosynthesis. Polyamines such as Spm could regulate ethylene biosynthesis through inhibition of ACC synthase [84], and ethylene is a direct inhibitor of arginine decarboxylase (ADC) and SAMDC [85]. The application of spermine effectively inhibited ethylene production in maize under drought stress [52].

Brassinosteroids are phytohormones which contribute to robust plant growth and development and participate in biotic and abiotic stress modulations either individually or in combination with other phytohormones, such as ABA, auxin, cytokinins, ethylene, jasmonic acid, salicylic acid, and gibberellins. A significant relationship between brassinosteroids and polyamines was evident from a study showing that epibrassinolide treatment enhanced polyamine levels, which in turn mitigated copper stress [86]. Treatment with a brassinosteroid derivative preserved the polyamine level similar to non-stress conditions and reduced the effect of salt stress.

Salicylic acid (SA) is a signaling molecule with wide activity, which can enhance defense systems in plants in stressful environments. Only a few studies have examined the dynamic interaction of SA and PAs under stress conditions. However, recent studies have revealed that SA treatment affects the synthesis and/or catabolism of polyamines [87,88]. Seed priming with spermine has a strong effect on endogenous content of salicylic acid in wheat under salt stress [89]. According to the above findings, we assume that there is a close link between SA content and polyamines, but the details underlying this relationship under stress conditions need clarification.

## 6. Omics Strategies for Using Spermine to Reduce Drought-Induced Oxidative Stress

An integration of bioinformatics approaches focusing on the genome, transcriptome, proteome, and metabolome is essential for developing a workable strategy for improving drought tolerance through optimization of spermine biosynthesis. Very few researchers have published work on those approaches for maximizing spermine production and efficient use in plants under water deficit conditions. The relevant mechanisms still need to be clarified. However, some recent studies utilized multi-omics approaches to explain spermine’s role in reducing drought stress. In addition, the availability of genome sequences and relevant databases enabled us to combine multi-omics methods to determine relative gene expression levels on a large scale. In the following section, we consider the efficacy of transcriptomic and proteomic methods in optimizing spermine activity.

### 6.1. Transcriptomics

Recent studies have revealed that Spm is involved in root growth and development, brassinosteroid signaling, ABA-dependent pathways, nonspecific phospholipase action, and the tolerance to abiotic stresses [70]. Spm enhanced phospholipase C activity, indicating greater interaction with phosphoinositide-Ca^2+^ signaling [90]. Spm also regulated Ca^2+^ homeostasis as well as upregulation of Ca^2+^ signaling genes [49]. Cloning and transfer of Spm biosynthetic genes derived from various sources demonstrated a remarkable improvement in environmental stress tolerance in numerous transgenic plants [72]. Many recent studies showed that the elevation of Spm in transgenic plants through overexpression of *ADC*, *SPMS*, and *SAMDC* genes improved drought tolerance. The following table shows those genes involved in protecting plants from drought stress (Table 2).

Overexpression of *SMDC1* or *SPMS* genes in *Arabidopsis* has been found to elevate Spm production [76,98]. However, in abiotic stress, *ADC* gene expression also plays a pivotal role in polyamine production. Transcription factors such as ABF, MYB, and WRKY have been implicated in *ADC* gene regulation [99]. Overexpression of Spm biosynthetic genes produced higher concentrations of putrescine under stress conditions, thereby promoting Spm synthesis and protecting plants from drought. Momtaz et al. 2010 [94] isolated and incorporated the *ScSAMDC* gene from *Saccharomyces cerevisiae* into two Egyptian cotton varieties (Giza 88 and Giza 90) and measured a significant increase in Spm accumulation and drought tolerance in both transgenic varieties. Another study showed that enhancing arginine decarboxylase (ADC) expression in transgenic plants resulted in elevated Put, Spd, and Spm levels, and protected plants from water deficit [74]. A significant increase in expression of genes related to ABFs and HSPs has been reported in Spm pre-treated trifoliate orange seedlings under high temperature and drought stress [16].

In a transcriptomic analysis study, upregulation of 1886 genes and downregulation of 2301 genes was observed in plants overexpressing SAMDC1, and upregulation of 907 genes and downregulation of 1648 genes was found in plants overexpressing spermine synthase (SPMS). Between the two groups, 233 upregulated genes and 328 downregulated genes were common. Both SAMDC and SPMS demonstrated involvement of the osmotic stress-responsive genes, ABA, Ca^2+^, JA, and SA. Overproduction of Spm in plants upregulated 23 RLKs, 3 MAPKs, and 7 Ca^2+^-regulating genes [76]. The evidence presented in these recent transcriptomic studies suggests that Spm induces drought tolerance in plant though ABA, JA, and Ca^2+^ signaling pathways.

### 6.2. Proteomics

Recent advanced proteomics approaches have been used for characterizing and sorting the complex structures and interactions of proteins in various kinds of cells. These methods can be utilized to deliver information relating to the proteomes, protein interaction maps, and protein localizations related to stress signaling and stress tolerance [100]. For decades, a variety of techniques such as microarrays, 2-DE, HPLC, and mass spectrometry have been widely used for proteome analysis. Proteomics methods have not been as often applied to studies of Spm-induced drought stress tolerance compared to the more widely used transcriptomics approaches. Thus, translational and post-translational proteomics studies could reveal information about protein interactions involved in Spm metabolism and their mode of action in enhancing drought tolerance.

In a proteomic study, 2-DE and MALDI-TOFMS analysis revealed 54 proteins to be associated with drought tolerance [101]. In another study, expression of 11 proteins related to photosystem, light reaction, glycolysis, nucleotide metabolism, and Calvin cycle (U1-U3 and U5-U12) was increased and expression of 23 proteins mostly related to photosystem, Calvin cycle, glyoxylate cycle, redox regulation (D1-D4, D6-D20, and F1-F4) was decreased after exogenous application of Spm. Among the proteins significantly upregulated were U5, U7, U9, and U12, which are involved in photosynthesis and amino acid and nucleotide metabolism. Nucleoside diphosphate kinase (NDPK) was closely associated with Spm-induced antioxidant activity during stress. Antioxidant enzymes like 2-Cys POD, APX, and Cu/Zn SOD were also upregulated by Spm [68]. These proteomics studies suggest a possible role of photosynthesis, amino acid and nucleotide metabolism, and stress-responsive enzymes in enhancement of drought tolerance in plants with high levels of Spm. However, the above proteomics studies on the beneficial effects of Spm on plants are still incomplete. Further advanced proteomics techniques could be useful in developing strategies for using Spm to enhance drought tolerance in plants.

## 7. Future Prospects and Conclusions

Drought negatively affects root development, and consequently impairs the growth of the upper parts resulting in decreased global crop yields. Thus, it is crucial to mitigate drought stress and develop drought stress-tolerant cultivars to ensure food security. Many studies confirm that Spm levels are increased under drought stress in plants, and this plays an important role in physiological functions. Spm activates antioxidants and promotes ROS scavenging under drought stress to protect biomolecules and membranes from damage. It also plays an important role as a signaling molecule and interacts with nitric oxide and phytohormones to enhance stress tolerance. As a result, Spm application has been gaining in popularity for improving drought tolerance in plants. However, there are still many exciting questions that need to be resolved in the future, to enrich our understanding of the mechanisms involved. Which transcription factors are involved in Spm-induced defense gene activation? Which regulatory mechanisms control Spm-mediated oxidative homeostasis under drought conditions? Answers to these questions and others will provide useful knowledge about Spm’s role in plant physiology, which will help to fill the gaps in our molecular toolkit. The application of a multidisciplinary approach benefiting from molecular techniques, selective breeding, and new biotechnology strategies is necessary to fully unlock the significant role of Spm in plant stress management to achieve sustainable crop production throughout the world.

## Figures and Tables

**Table 1:** Spermine mediated growth, improved photosynthetic parameters and osmoregulation, and enhanced antioxidant defense in different plant species under drought stress.

Species	Stress	Spermine Treatment	Effect	Outcome	References
*Arabidopsis thaliana*	Drought stress (1/2 MS agar plates)	1 mM (exogenous pretreated seedlings)	Enhanced chlorophyll content, potential role in stomatal movement	Spm protected against drought stress	[49]
*Cynodon dactilon*	Drought stress (withholding water)	5 mM (exogenous)	Proteins involved in ROS balance stimulated by spermine. Energy-related pathways stimulated by Spm treatment	Improved drought stress tolerance	[47]
Cucumber	Drought	1 mM (pretreated seed)	Reduced ion leakage from the membrane and less lipid peroxidation	Nitric oxide acts downstream of Spm during drought stress to enhance stress tolerance	[50]
Creeping Bentgrass (Penn G2)	Drought (withholding water)	1 mM (exogenous)	Spermine-treated plants maintained significantly higher turf quality (TQ), relative water content (RWC), and photochemical efficiency	Protected creeping bentgrass from drought stress	[48]
Chinese dwarf cherry (*Cerasus humilis*)	Drought stress (withholding water)	0.2 mM (exogenous)	Increased RWC and prevented lipid peroxidation	Prevented drought-induced oxidative damage	[51]
Lettuce	Drought (10% polyethylene glycol, PEG)	0.1 mM (exogenous)	Increased plant height and root length. Upregulated antioxidant activity	Significantly alleviated drought stress	[11]
Maize	Drought (50% and 75% field capacity)	25 mgL (exogenous)	Increased content of protein, phenolic, flavonoids, and amino acids	Improved drought tolerance by increasing ethylene and polyamine synthesis	[52]
Maize (Giza 10 and Giza 129 cultivars)	Drought (50% and 75% field capacity)	25 mgL (exogenous)	Stimulated synthesis of antioxidant enzymes, and promoted ROS scavenging	Enhanced drought tolerance and reduced ROS accumulation	[53]
Mung bean (*Vigna radiata* L. cv. BARI Mung-2)	Combined drought and high temperature stress	0.2 mM (exogenous pretreated seedlings)	Upregulated antioxidant enzymes. Reduced methylglyoxal toxicity by stimulating glyoxalase systems	Improved tolerance to drought and high temperature stress	[29]
Orange (*Poncirus trifoliata* [L.] Raf.)	Combined heat and drought	1 mmol L-1 (exogenous pretreated seedlings)	Activated antioxidant enzymes such as CAT, SOD, and peroxidases; induced heat shock proteins and abscisic acid-response element binding factors	Enhanced drought and heat tolerance in a perennial fruit crop	[16]
*Oryza sativa*	Drought (50% field capacity)	10 µM (seed priming treatments and foliar application)	Activated antioxidant enzymes. Enhanced ROS scavenging and stress-related gene expression	Enhanced drought and heat tolerance in rice seedlings	[54]
Red tangerine (*Citrus reticulata* Blanco)	Drought (MS agar plates)	1 mM (pretreated seed)	Increased enzymatic antioxidant activity such as SOD and peroxidase and ROS scavenging	Prevented oxidative damage and increased drought tolerance	[55]
*Rosa damascena* Miller var. trigintipetala Dieck	Drought (50% and 100% field capacity)	0.5 mM (exogenous)	Improved growth (RWC), photosynthetic pigments and stomatal conductance(gs)	Mitigated drought stress	[56]
Soybean cultivars (Giza 111 and Gazi 21)	Drought (0, −0.1, −0.5, and −1.1 MPa)	0.2 mM (pretreated seed)	Pigment enhancement, membrane stabilization, osmolyte accumulation, and water balance	Increased drought tolerance of soybean cultivar	[10]
Soybean	Drought (9% PEG)	0.2 mM (exogenous)	Enhanced CAT, SOD, and POD activities; reduced lipid peroxidation	Improved drought tolerance of soybean	[57]
Valerian	Drought (withholding water)	0.1 mM (exogenous)	Increased photosynthetic pigments and antioxidant enzyme activity	Improved drought tolerance	[58]
Wheat	Drought (withholding water)	100 µM (exogenous)	Increased photosynthetic pigments, antioxidants, and Rubisco	Enhanced drought tolerance of wheat by reduction of oxidative injury	[9]
Wheat	Drought (withholding water)	100 µM (exogenous)	Increased cell water status and accumulation of osmoprotectants	Improved drought tolerance	[32]
Wheat	Drought (soil water potential at −60 ± 5 kPa)	1 mM (exogenous)	Relieved inhibition caused by drought stress	Enhanced grain filling and drought resistance	[44]
White clover	Drought stress (20% PEG 6000)	0.5 mM (exogenous)	Improved sugar metabolism and dehydrin biosynthesis	Mitigated drought stress	[33]

**Table 2:** Spermine biosynthetic genes involved in tolerance to drought and other abiotic stresses in plants.

Gene	Source	Transgenic Plant	Abiotic Stress Tolerance	References
*ADC*	*Datura stramonium*	*Oryza sativa*	Drought	[68]
*ADC*	*Avena sativa*	*Solanum meloangena*	Drought, high temperature	[91]
*ADC*	*Avena sativa*	*Triticum aestivum*	Drought	[92]
*SAMDC*	*Datura stramonium*	*Oryza sativa*	Drought	[93]
*SAMDC*	*Saccharomyces cerevisiae*	Egyptian cotton varieties. Giza 88, Giza-90	Drought	[94]
*SAMDC*	*Saccharomyces cerevisiae*	*Solanum lycopersicum* cv. Pusa Ruby	Drought, Salt	[95]
*SAMDC*	*Sesamum indicum*	*Nicotiana tabacum*	Drought	[96]
*SPMS*	*Pyrus bretschneideri*	*Arabidopsis thaliana*	Drought, Salt	[97]

## Data Availability

No new data were created or analyzed in this study. Data sharing is not applicable to this article.
